# Association of accelerated body mass index gain with repeated measures of blood pressure in early childhood

**DOI:** 10.1038/s41366-019-0345-9

**Published:** 2019-04-02

**Authors:** Karen M. Eny, Jonathon L. Maguire, David W. H. Dai, Gerald Lebovic, Khosrow Adeli, Jill K. Hamilton, Anthony J. Hanley, Muhammad Mamdani, Brian W. McCrindle, Mark S. Tremblay, Patricia C. Parkin, Catherine S. Birken, for the TARGet Kids! Collaboration

**Affiliations:** 1 Child Health Evaluative Sciences, The Hospital for Sick Children, Toronto, Canada; 2 Applied Health Research Centre, Li Ka Shing Knowledge Institute, St. Michael’s Hospital, Toronto, Canada; 3 Department of Pediatrics, St. Michael’s Hospital, Toronto, Canada; 4 Institute of Health Policy, Management and Evaluation, Dalla Lana School of Public Health, University of Toronto, Toronto, Canada; 5 Department of Pediatrics, Faculty of Medicine, University of Toronto, Toronto, Canada; 6 Department of Nutritional Sciences, University of Toronto, Toronto, Canada; 7 Department of Laboratory Medicine and Pathobiology, Faculty of Medicine, University of Toronto, Toronto, Canada; 8 Division of Endocrinology, The Hospital for Sick Children, Toronto, Canada; 9 Li Ka Shing Centre for Healthcare Analytics Research and Training, St. Michael’s Hospital, Toronto, Canada; 10 Preventative Cardiology, The Hospital for Sick Children, Toronto, Canada; 11 Children’s Hospital of Eastern Ontario Research Institute, Ottawa, Canada

**Keywords:** Risk factors, Cardiovascular diseases

## Abstract

**Background/objectives:**

We examined the association for rates of age- and sex-standardized body mass index (zBMI) gain between 0–3, 3–18, and 18–36 months with BP in children at 36–72 months of age.

**Methods:**

We collected repeated measures of zBMI and BP in 2502 children. zBMI was calculated using the World Health Organization standards. Each child’s zBMI at birth and rates of zBMI gain in each period from birth to 36 months were estimated using linear spline multilevel models. Generalized estimating equations were used to determine whether zBMI at birth and zBMI gain between 0–3, 3–18, and 18–36 months were each associated with repeated measures of BP at 36–72 months of age. We sequentially conditioned on zBMI at birth and zBMI gain in each period prior to each period tested, as covariates, and adjusted for important socio-demographic, familial, and study design covariates. We examined whether these associations were modified by birthweight or maternal obesity, by including interaction terms.

**Results:**

After adjusting for all covariates and conditioning on prior zBMI gains, a 1 standard deviation unit faster rate of zBMI gain during 0–3 months, (β = 0.59 mmHg; 95% CI 0.31, 0.86) and 3–18 months (β = 0.74 mmHg; 95% CI 0.46, 1.03) were each associated with higher systolic BP at 36–72 months. No significant associations were observed, however, for zBMI at birth or zBMI gain in the 18–36 month growth period. zBMI gains from 0–3 and 3–18 months were also associated with diastolic BP. Birthweight significantly modified the relationship during the 3–18 month period (*p* = 0.02), with the low birthweight group exhibiting the strongest association for faster rate of zBMI gain with higher systolic BP (β = 1.31 mmHg; 95% CI 0.14, 2.48).

**Conclusions:**

Given that long-term exposure to small elevations in BP are associated with subclinical cardiovascular disease, promoting interventions targeting healthy growth in infancy may be important.

## Introduction

Blood pressure (BP) in childhood tracks into adulthood [1] and persistent exposure to elevated levels in childhood and adulthood is associated with increased risk of carotid artery atherosclerosis [2]. Prevalence estimates from the National Health and Nutrition Examination Survey (NHANES) have reported that 1 in 10 children have elevated BP (≥90th percentile [3]) [4]. Identifying risk factors associated with BP in early childhood (0–6 years) is important to develop targets for cardiovascular disease (CVD) prevention.

Rapid postnatal growth has been proposed as an important predictor of elevated BP. While some studies have supported the association between accelerated weight or adiposity gain in infancy with higher BP in line with the “growth acceleration hypothesis” [5–8], faster growth rates during the period in closest proximity to BP measurement has been reported to be more strongly associated with BP in childhood [5, 7, 9–13]. However, the growth period in closest proximity to BP varies in age range and duration of time over which growth is measured within and across prior studies [5, 9, 12, 13], which challenges the interpretation of whether the last period represents a “sensitive period” [14]. In addition, the effect of birthweight on the relationship between accelerated growth and BP in childhood remains unclear [7, 9, 11, 12]. This may be attributable to the exclusion of children with low birthweight [12], or relatively small sample size of previous studies [9]. Maternal obesity status is also an important early life risk factor associated with rapid growth as well as childhood BP [15], but it is unknown whether maternal obesity status modifies the relationship between rapid growth and BP in early childhood.

BP is known to exhibit high within-person variability. Repeated measures of BP taken over two or more visits have been shown to improve the reliability of BP measurement in children, as well as the correlation between childhood and adulthood measures of BP [16, 17]. To our knowledge no study has yet examined the relationship between rate of age- and sex-standardized body mass index (zBMI) gain with repeated measures of BP in early childhood.

The primary objective of this study was to determine whether rates of zBMI gain between 0–3, 3–18, and 18–36 months were associated with repeated measures of BP in children at 36–72 months of age. The secondary objectives included determining whether birthweight and/or maternal obesity modify the relationship between rates of zBMI gain and BP. We also determined whether accelerated zBMI gain in each period was associated with elevated BP according to the 2017 American Academy of Pediatrics (AAP) normative pediatric tables.

## Subjects and methods

### Study design and participants

We conducted a longitudinal study using repeated measures of zBMI and BP in children participating in the TARGet Kids! (The Applied Research Group for Kids) cohort [18]. TARGet Kids! is an ongoing longitudinal study and the largest practice-based research network in Canada, whereby children are recruited and followed prospectively during well-child visits across primary care practices in Toronto and Montreal, Canada. Overall, 7997 healthy children were recruited from July 2008 to July 2017 across thirteen TARGet Kids! clinics. We used two analytical stages for this study; Stage 1 modeled repeated measures of zBMI between 0–3, 3–18, and 18–36 months of age to generate rates of zBMI gain for each child in each period. Stage 2 modeled the association between rates of zBMI gain with BP in children at 36–72 months of age. Supplementary Figs. 1 and 2 outline the participant flow for stage 1 and stage 2. Since Stage 1 models zBMI gains for each child using the World Health Organization (WHO) Growth Standards, which did not include preterm infants or very low birthweight infant [19], children born <37 weeks of gestational age (*n* = 741 stage 1; *n* = 476 stage 2) and/or children with missing or very low birthweight (<1 kg) were excluded (*n* = 464 stage 1; *n* = 422 stage 2). After all inclusion/exclusion criteria were applied, 4258 children (with 26 843 zBMI observations) were included in stage 1 and 2502 of these children (with 4963 BP observations) were included for analysis in stage 2. Approval was obtained from the Research Ethics Boards at the Hospital for Sick Children and St. Michael’s Hospital and parents of participating children provided written informed consent.

### Weight, height/length and zBMI

Weight and length/height were measured during well-child visits by trained research assistants embedded in each primary care practice (21,556 observations from 7931 children, Supplementary Fig. 1). A baby scale was used to measure weight in children <2 years of age, and a precision digital scale (SECA model 703, measurement accuracy ±0.025%; SECA, Hamburg, Germany) was used for older children. A calibrated length board was used to measure length in children <2 years of age and a calibrated stadiometer (SECA, Germany) was used to measure height of older children. Repeated measures of weight and length/height were also abstracted from electronic health records from routine physician visits when available for 4063 of the children (observations = 34 800). We accounted for whether the zBMI observation was obtained from research vs routine visits (visit type) as a covariate. We have reported coefficients of reliability [based on calculated technical error of measurement [20]] of >99% for both intra- and interobserver comparisons for length, height, and weight comparing research with routine anthropometric measures in three of our TARGet Kids! clinics (S Carsley and CS Birken, unpublished data, 2019). To avoid overweighting visits that were not well-child physician visits, we randomly removed anthropometric observations (8.4% of routine physician visit observations) measured during routine physician visits that occurred close together in time (Supplementary methods) [13]. BMI was calculated and standardized according to the WHO growth standards, using the child’s exact age (in months) that the weight and height were measured, and subtracting each child’s BMI from the sex-specific mean and dividing by the sex-specific SD at each age using the igrowup package for SAS. We chose to standardize BMI using the WHO growth standards to be able to describe growth in our cohort relative to an external reference. This approach also adequately accounts for changes in growth rates with age and ensures constant variance as children age [14]. We used the WHO cutpoints of <−5.0 and >+5.0 SD-units to identify and evaluate potentially implausible z-scores of height, weight and BMI [21, 22], and set the values to missing as described in Supplementary methods (0.5% of observations) [23]. Children with at least two measures of zBMI from birth to 36 months were included in this stage, however, we also performed a sensitivity analysis to compare stage 1 results when including children with at least one zBMI observation.

### Blood pressure

Systolic (SBP) and diastolic (DBP) blood pressure were measured by trained research assistants once per year at each clinic when the children were 3–6 years of age according to the Fourth Report guidelines [3]. BP measurements were also available from routine well-child physician visits (1426 routine; 3537 research visit observations), and measured using the same approach. An appropriate sized cuff was used in each clinic to measure BP once per visit, as usually done in clinical practice, by auscultation, on the child’s upper arm. To determine biological plausibility of BP measures we used upper cutpoints of >260 mmHg for SBP and >200 mmHg for DBP [24]. No values were greater than 180 mmHg in our cohort; therefore, no upper limit BP values were excluded. We excluded SBP and DBP <50 mmHg and <25 mmHg, respectively, as these represent values in the hypotensive range (observations = 4) [25]. The 2017 AAP Clinical Practice Guideline were used to define elevated BP as ≥90th percentile for age-, sex-, and height-standardized SBP or DBP values [26].

### Statistical analysis

#### Stage 1: Rates of zBMI Gain

We used linear spline multilevel models [27] to calculate rates of zBMI gain using proc MIXED in SAS, modeling age as a random effect using an unstructured covariance. We fit knot points at 3 and 18 months, which were previously identified in this cohort as turning points for changes in zBMI trajectories using a loess curve [23]. The resulting model provided an estimate of the average zBMI at birth (intercept) and the average rate of zBMI gain (splines/slope) between 0–3, 3–18, and 18–36 months (Supplementary Table 1). By specifying unstructured random effects for the intercept and slope (splines), zBMI at birth and zBMI trajectories were estimated for each child by adding the random intercept and splines for each child to the estimates of the population mean zBMI at birth (β_0_) and rates of zBMI gain (β_1_–β_3_) for each period (0–3, 3–18, and 18–36 months) [28]. The stage 1 analysis included age and splines as predictors and adjusted for sex, and visit type (zBMI obtained from research vs routine visits). The estimated zBMI at birth and rates of zBMI gain during each period then served as the predictor variables for each child in stage 2. To provide a clinically meaningful interpretation of the effect size and to compare effect estimates across age periods [14], we standardized the rates of zBMI gain to have a mean of 0 and SD 1, using the mean ± SD of zBMI growth rates from Supplementary Table 1.

#### Stage 2: Association of rates of zBMI gain with BP

We used generalized estimating equations (GEE) to test the association between zBMI at birth and rates of zBMI gain between 0–3, 3–18, and 18–36 months of age with repeated measures of SBP and DBP at 36–72 months of age. GEE allows for children with one or more measures of blood pressure to be included in the analysis and accounts for within-person correlation among children who had repeated measures of blood pressure available between the ages of 36–72 months of age. In our unadjusted and adjusted analyses we sequentially conditioned on zBMI at birth and zBMI gain in each period prior to each period tested, as covariates in the model. This was done to test the effect of zBMI gain in each period independent of prior body size. We then fit a second model, building on the unadjusted (conditioned) model adjusting for the following covariates selected *a* priori: age and height at time of BP measures, sex, family income, maternal (education, ethnicity, BMI, hypertension during pregnancy), parental history of hypertension, breastfeeding duration (not included in the model for zBMI at birth), visit type (research vs routine), and clinic. Please see the Supplementary methods for details on how each covariate was measured.

To determine whether associations for zBMI gain and BP differed by sex we tested for a sex∗zBMI at birth and a sex∗zBMI gain interaction in each period. We similarly tested for interaction by birthweight and maternal obesity status in each model to examine effect modification. Logistic regression analysis with GEE (exchangeable covariance structure) was adjusted for the same covariates (except for sex, age and height) as in the linear regression GEE to determine whether zBMI at birth and zBMI gains were associated with elevated BP (≥90th percentile AAP criteria) [26].

We used multiple imputation to impute missing covariates. Proportion of missing values for all covariates was <16%. Models were run on 10 imputed datasets using the mice package in R. Sensitivity analyses were performed for subjects who had all covariates available (complete case analysis). In addition, children with missing gestational age (23%) were assigned as full-term in our analyses and a sensitivity analysis excluding children with missing gestational age was performed. Results from sensitivity analyses were similar to our main findings. Residuals were examined to evaluate adequate model fit (data available upon request). All p-values are two-tailed and not adjusted for multiple comparisons. *P*-values <0.05 were considered statistically significant. SAS v9.4 (SAS Institute, Cary, NC) and R v3.4.1 (R:https://www.r-project.org/) were used for all analyses.

## Results

Participant characteristics of the 2502 children included in the stage 2 analyses examining the association between zBMI gain and BP are shown in Table 1. The majority of children (60%) had ≥2 measures of BP from 36–72 months of age (Supplementary Table 2). The average age at the first measure of BP was 44 months (Table 1). Average SBP was 87 mmHg and 1.8% of children had elevated SBP. Average DBP was 56 mmHg and 17% of children had elevated DBP.

**Table 1:** Participant characteristics

	*n*	Mean (SD) or *N* (%)^a^
Age (months)^b^	2502	44.0 (8.7)
SBP (mmHg)^b^	2502	87 (7.9)
Elevated SBP^b,c^	2494	
No		2449 (98.2)
Yes		45 (1.8)
DBP (mmHg)^b^	2502	56 (7.5)
Elevated DBP^b,c^	2494	
No		2064 (82.8)
Yes		430 (17.2)
Height (cm)^b^	2494	100.9 (8.2)
Weight (kg)^b^	2496	16.4 (4.1)
zBMI^b^	2485	0.35 (1.4)
Birthweight (kg)^d^	2502	3.39 (0.51)
<2.5 kg		110 (4.4)
≥2.5 and <4 kg		2112 (84.4)
**≥**4 kg		280 (11.2)
Breastfeeding duration (months)^e^	2321	11.1 (7.9)
Maternal age (years)^d^	2374	36.0 (4.5)
Maternal ethnicity^d^	2351	
European		1718 (73)
East Asian		144 (6)
South/Southeast Asian		180 (8)
Other		309 (13)
Income^d^	2089	
$0–$39,999		123 (5.9)
$40,000–$79,999		262 (12.5)
$80,000–$149,999		684 (32.7)
$150,000+		1020 (48.8)
Maternal education^d^	2433	
College/University		2215 (91)
High school		204 (8)
Public school		14 (1)
Maternal BMI^d^	2283	24.6 (4.6)
BMI <30		2037 (89)
BMI ≥30		246 (11)
Maternal smoking during pregnancy^d^	2301	
No		2272 (99)
Yes		29 (1)
Gestational high BP^d^	2358	
No		2273 (96)
Yes		85 (4)
Family history of hypertension^d^	2412	
No		2327 (96.5)
Yes		85 (3.5)

^a^Based on complete case analysis

^b^First visit when BP was measured

^c^Defined as SBP ≥90th percentile in the 2017 AAP normative BP tables

^d^Collected at enrollment

^e^Determined using information from the most recent visit for each child

Supplementary Table 1 shows the estimated rates of zBMI gains from modeling the repeated measures of zBMI from birth to 36 months of age in Stage 1. Each child contributed a median of seven repeated measures of zBMI (interquartile range = 3–9 measures) observations from birth to 36 months of age (*n* = 4258, observations = 26 843), with 84% of children having three or more repeated measures of zBMI. The estimated rates of zBMI gain in the first 3 months was −0.12 zBMI-units per month, followed by an acceleration of 0.06 zBMI-units per month during 3–18 months, and a slower acceleration 0.01 zBMI-units per month in the last period (18–36 months). We [23] and others [29–32] have shown similar patterns of zBMI deceleration and gains using the WHO growth standards. Supplementary Table 3 shows overall good model fit for stage 1 growth trajectories with the 95% limits of agreement for the observed vs predicted zBMI values falling within 10% of the mean zBMI from birth to 36 months. A sensitivity analysis of Stage 1 was performed with very strong correlations (>0.93) of estimates of the zBMI at birth and zBMI gains in the three periods in children with at least one zBMI measure, compared to at least two zBMI measures (Data available upon request).

Table 2 shows the association between zBMI at birth and rates of zBMI gain in each period with BP. zBMI at birth was not associated with SBP or DBP at 36–72 months of age. Rates of zBMI gain between 0–3 months was associated with higher BP, such that a 1 SD unit faster rate of zBMI gain was associated with a 0.50 mmHg higher SBP in the unadjusted model, and 0.59 mmHg higher SBP in the adjusted analysis (*p* < 0.001). In the 3–18 months period, a 1 SD unit faster rate of zBMI gain was associated with a 1.15 mmHg higher SBP (*p* < 0.001) in the unadjusted model, and a 0.74 mmHg higher SBP (*p* < 0.001) in the adjusted analysis. In the 18–36 months period, a 1 SD unit faster rate of zBMI gain was associated with a 0.61 mmHg higher SBP in the unadjusted model (*p* = 0.02); however, the effect was attenuated and no longer significant after adjusting for covariates. Table 2 also shows results for DBP. Overall, we report similar associations in the adjusted analyses as observed for SBP, but with smaller effect sizes. To determine whether significant associations remained with BP independent of concurrent zBMI, we additionally adjusted for zBMI at the time BP was measured. We observed a slight attenuation in effect estimates, but the overall patterns observed in the main analyses remained (Supplementary Table 4).

**Table 2:** Association of zBMI at birth and rate of zBMI gain in each period with blood pressure (mmHg)

	Systolic blood pressure				Diastolic blood pressure			
	Unadjusted^b^		Adjusted^b,c^		Unadjusted^b^		Adjusted^b,c^	
Period (months)^a^	β	95% CI	β	95% CI	β	95% CI	β	95% CI
zBMI at birth^d^	−0.04	(−0.31, 0.23)	0.04	(−0.20, 0.28)	0.06	(−0.21, 0.32)	0.15	(−0.08, 0.37)
zBMI gain 0–3 m^e^	0.5	(0.19, 0.80)	0.59	(0.31, 0.86)	0.17	(−0.12, 0.45)	0.28	(0.04, 0.53)
zBMI gain 3–18 m	1.15	(0.85, 1.45)	0.74	(0.46, 1.03)	0.62	(0.35, 0.88)	0.44	(0.20, 0.68)
zBMI gain 18–36 m	0.61	(0.10, 1.12)	0.43	(−0.06, 0.92)	−0.7	(−1.18, −0.22)	−0.03	(−0.42, 0.37)

^a^Each row shows results from four separate models

^b^zBMI gain is sequentially conditioned on zBMI at birth and rate of zBMI gain in each period occurring prior to the current period

^c^Adjusted for age and height at time of BP measures, sex, family income, maternal (education, ethnicity, BMI, hypertension during pregnancy), parental history of hypertension, breastfeeding duration, visit type, and clinic

^d^Not adjusted for breastfeeding duration

^e^Since we observed a deceleration of −0.12 zBMI-units per month in the first 3 months (Supplementary Table 1), the interpretation of the positive effect estimate shows that a 1SD-unit slower rate of decrease in zBMI-units from 0–3 months was associated with higher BP (mmHg)

**Fig. 1:**
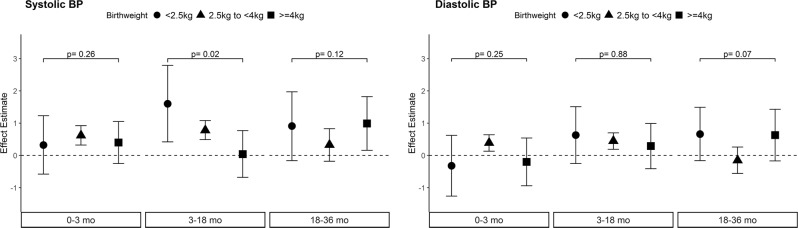
Effect estimate (95% CI) between zBMI gain in each period with BP by birthweight (p_interaction_ shown). BP blood pressure, mo months

Results from the analyses including the sex interaction term showed that the association between zBMI at birth and rates of zBMI gain with SBP and DBP were similar in boys and girls, with the exception of the association between accelerated zBMI gain in the last period with higher SBP (p_interaction_ = 0.03), where a stronger association was observed in girls (Supplementary Fig. 3). Birthweight significantly modified the relationship for accelerated zBMI gain during 3–18 months and SBP (p_interaction_ = 0.02), with the strongest positive relationship among low birthweight children (Fig. 1). Overall, maternal obesity status did not significantly modify the relationship between zBMI at birth or rates of zBMI gain during any period with BP (Supplementary Fig. 3).

Table 3 reports the association between zBMI at birth and accelerated zBMI gain in each period with odds of elevated SBP and DBP. In the first 3 months, a 1 SD unit faster rate of zBMI gain was associated with a 1.55-fold higher odds of elevated SBP (95% CI 1.18, 2.04). In the 3–18 months period, a 1 SD unit faster rate of zBMI gain was associated with a 1.42-fold higher odds of elevated SBP (95%CI 1.07, 1.89). Rate of zBMI gain during 18–36 months was not associated with odds of elevated SBP, but a 1 SD unit faster rate of zBMI gain in this period was associated with a 1.22-fold higher odds of an elevated DBP (95%CI 1.04, 1.44).

**Table 3:** Odds ratio (95% CI) for the association of zBMI at birth and rate of zBMI gain in each period with elevated blood pressure (mmHg)

	Systolic blood pressure				Diastolic blood pressure			
	Unadjusted^b^		Adjusted^b,c^		Unadjusted^b^		Adjusted^b,c^	
Period (months)^a^	Odds ratio	95% CI	Odds ratio	95% CI	Odds ratio	95% CI	Odds ratio	95% CI
zBMI at birth^d^	0.85	(0.66, 1.11)	0.92	(0.71, 1.20)	1	(0.90, 1.11)	1.01	(0.92, 1.11)
zBMI gain 0–3 m^e^	1.14	(0.83, 1.54)	1.55	(1.18, 2.04)	1.03	(0.93, 1.14)	1.07	(0.97, 1.18)
zBMI gain 3–18 m	1.91	(1.47, 2.49)	1.42	(1.07, 1.89)	1.02	(0.92, 1.13)	1.04	(0.94, 1.15)
zBMI gain 18–36 m	0.97	(0.62, 1.50)	0.64	(0.39, 1.05)	1.11	(0.92, 1.33)	1.22	(1.04, 1.44)

^a^Each row shows results from four separate models

^b^zBMI gain is sequentially conditioned on zBMI at birth and rate of zBMI gain in each period occurring prior to the current period

^c^Adjusted for family income, maternal (education, ethnicity, BMI, hypertension during pregnancy), parental history of hypertension, breastfeeding duration, visit type, and clinic

^d^Not adjusted for breastfeeding duration

^e^Since we observed a deceleration of −0.12 zBMI-units per month in the first 3 months (Supplementary Table 1), the interpretation of the positive effect estimate shows that a 1SD-unit slower rate of decrease in zBMI-units from 0–3 months was associated with a higher odds of elevated BP

## Discussion

In this study of 2502 children we show that accelerated zBMI gain in early (0–3 months) and late (3–18 months) infancy are each associated with higher levels of SBP and DBP at 36–72 months of age, independent of zBMI at birth and zBMI growth in prior periods. Among children born with low birthweight, faster rates of zBMI gain during the 3–18 month growth period were associated with a greater increase in BP. To our knowledge this is the first study that examined the association between rates of zBMI gain with BP measured repeatedly over multiple visits in early childhood.

Our findings are consistent with the study by Tilling et al. who also modeled growth rates estimated from linear spline multilevel models in the PROBIT cohort and demonstrated that faster weight gain in early (0–3 months) and late infancy (3–12 months), conditioned on prior body size, were each associated with higher BP in early childhood [12]. Here, we extend their work by modeling zBMI in order to characterize gains in adiposity independent of increases in height. In contrast, other studies have shown that rapid growth in early (0–6 months), but not late infancy (6–12 months), was associated with higher BP levels in childhood [5, 7]. Differences in findings across studies may be due to smaller sample size or length of periods examined [5, 7, 14]. In addition, a simulation study [28] demonstrated that effect estimates for the association between growth rates and BP in studies, which calculate growth rates using the arithmetic difference in body size between two ages, may be biased towards the null [7].

In the model unadjusted for covariates, but conditioned on prior zBMI gains, we observed a weak positive association of accelerated zBMI gain during the last period (18–36 months of age) with higher BP at 36–72 months of age. Despite closer proximity to measures of BP, the association was no longer significant after adjusting for covariates. Our results differ from other studies, that report stronger associations between accelerated growth in the last age period with higher levels of BP [5, 7, 9–13]. We accounted for a number of important covariates including family history of hypertension, gestational hypertension and child height, which were not consistently adjusted for in prior studies [5, 7, 9–13]. In addition, the last period we examined from 18 to 36 months of age, precedes the average age for adiposity rebound [33], and therefore may represent a biologically less influential phase. Indeed, studies that extended the age period beyond 36 months to include the adiposity rebound period have observed significant associations between faster growth rates and higher BP [9–12].

We observed a significant interaction between birthweight and zBMI gain during 3–18 months of age. Consistent with our findings, observations from the EDEN cohort report that children who were born small-for-gestational age and experience the most rapid growth exhibited the highest levels of BP [34]. In contrast, previous studies measuring BP in early childhood have not detected a significant effect modification by birthweight. This may be attributable to smaller sample sizes [9]. In addition, prior studies measured BP during a single visit rather than over several visits [7, 9, 11, 12]. Low birthweight has been shown to be associated with higher long-term BP variability over time [35], therefore, accounting for within-person variability may be important when examining effect modification by birthweight.

The small effect size observed in our cohort are in line with previous studies reporting effect sizes of a 1 mmHg higher SBP in early childhood associated with faster growth [5, 6, 9, 11, 12]. According to the amplification hypothesis, the relatively small effect size observed in early childhood may increase as children age [36]. For example, Chiolero et al found that weight gain from 1–5.5 years of age was associated with a 1 mmHg higher SBP in girls at 5.5 years of age, but the effect size of the association for weight gain between 1–5.5 years of age increased to 4 mmHg when BP was measured at 9 years of age [11]. Accelerated zBMI gain in early and late infancy in our cohort was associated with a 1.4–1.5-fold higher odds of elevated SBP. Persistent exposure to higher BP beginning in early childhood may result in higher risk of CVD burden, as illustrated by greater carotid intima media thickness in the International Childhood Cardiovascular Cohort consortium [2]. The Cardiovascular Risk in Young Finns study has shown that SBP was 1.92 mmHg higher among adults with coronary artery calcium compared to those without, using long-term average BP measures over a 27-year period beginning in adolescence [37].

### Strengths and limitations

Unlike using conditional residuals or the arithmetic difference to model growth rates, linear spline multilevel models do not require that anthropometrics are measured at the same time for all subjects in the cohort [38]. Some prior studies have modeled growth rates using unstandardized body size [12, 13], however, in the study by Aris et al., no differences were reported between their analyses examining associations with BP using unstandardized and WHO-standardized BMI velocities. using unstandardized and WHO-standardized BMI velocities [9].

We used repeated measures of BP available in our study since children have been shown to exhibit particularly high within-person variability in BP [16]. A limitation of our study is that we measured BP only once per visit and therefore we are unable to remove additional random error associated with within-person fluctuations that occur on a given day. Random error associated with measuring BP may lead to increases in standard error, thus widening the confidence interval of the effect estimates [39]. In addition, use of a single BP measurement on a single occasion may have resulted in a higher estimate of elevated BP, and therefore effect estimates for the association with the odds of elevated BP should be interpreted with caution. However, when blood pressure classification change was examined among NHANES participants, 91% of children maintained their BP classification when comparing classification across three repeated measurements taken over one visit [40]. We used the new AAP age-, sex-, and height-standardized BP percentiles, which updated the 2004 tables by excluding children with overweight and/or obesity (zBMI ≥ 85th percentile) [26]. Given the lower threshold for elevated BP in the updated tables [26], this may have also contributed to higher prevalence estimates for elevated BP observed. We measured BP by auscultation in our cohort, which is advantageous in comparison to oscillometric devices which may overestimate BP, and facilitates comparison to the normative pediatric tables that were based on auscultation [3]. Auscultation measures, however, may be prone to interobserver bias and we therefore adjusted for clinic to account for this potential source of bias [16]. Finally, generalizability of results from our study may be limited to children with reported ethnicity as European-descent (73%) and generally high socioeconomic status, living in urban settings. However, we note that the distribution of ethnicity in our study is comparable to Canadian census data [41]. This study does not address rates of zBMI gain and BP in preterm infants, or infants born <1000 g, who may be at increased risk of elevated BP. Further studies are needed in other settings to establish external validity.

In conclusion, results from our study demonstrate that rates of zBMI gain from 0–3 months and 3–18 months of age are associated with higher blood pressure in early childhood, independent of zBMI at birth, and zBMI gains in prior periods. We observed a stronger association between rapid growth during 3–18 months of age with BP in children born with low birthweight, and therefore highlight a developmental window to prioritize interventions for children in this risk group. Further studies are needed to replicate our observations and to identify interventions to promote growth with optimal short-term and long-term health outcomes on CVD during these distinct periods of early life.

## Supplementary information

Supplementary Methods

Supplementary Table 1

Supplementary Table 2

Supplementary Table 3

Supplementary Table 4

Supplementary Figure 1

Supplementary Figure 2

Supplementary Figure 3

Supplementary Figure 3 Legend
