# Supplementary material for: Association of accelerated body mass index gain with repeated measures of blood pressure in early childhood
Source: Int J Obes (Lond). 2019 Apr 2;43(7):1354–62. doi: 10.1038/s41366-019-0345-9 (PMC6760600; doi:10.1038/s41366-019-0345-9)
Supplement: Supplementary file 1 — Supplementary Methods [file 41366_2019_345_MOESM1_ESM.docx]

# **Supplementary Figure 3 Legend**

Effect estimate (95% CI) from adjusted conditional models examining the association between zBMI gain in each period with BP by sex (top panels) and maternal obesity (bottom panels) are shown along with p_interaction_ for each factor. BP: blood pressure; mo: months.
