# Supplementary material for: Association of accelerated body mass index gain with repeated measures of blood pressure in early childhood
Source: Int J Obes (Lond). 2019 Apr 2;43(7):1354–62. doi: 10.1038/s41366-019-0345-9 (PMC6760600; doi:10.1038/s41366-019-0345-9)
Supplement: Supplementary file 2 — Supplementary Table 1 [file 41366_2019_345_MOESM2_ESM.docx]

**ONLINE SUPPLEMENT**

**Association of accelerated body mass index gain with repeated measures of blood pressure in early childhood**

Karen M. Eny^1^, Jonathon L. Maguire^2,3,4,5,6^, David W.H. Dai^2^, Gerald Lebovic^2,4^, Khosrow Adeli^7^, Jill K. Hamilton^5,8^, Anthony J. Hanley^6^, Muhammad Mamdani^9^, Brian W. McCrindle^1,5,10^, Mark S. Tremblay^11^, Patricia C. Parkin^1,4,5^, Catherine S Birken^1,4,5,6^, for the TARGet Kids! Collaboration

^1^Child Health Evaluative Sciences, The Hospital for Sick Children, Toronto, Canada

^2^Applied Health Research Centre, Li Ka Shing Knowledge Institute, St. Michael’s Hospital, Toronto, Canada

^3^Department of Pediatrics, St. Michael’s Hospital, Toronto, Canada

^4^Institute of Health Policy, Management and Evaluation, Dalla Lana School of Public Health, University of Toronto, Toronto, Canada

^5^Department of Pediatrics, Faculty of Medicine, University of Toronto, Toronto, Canada

^6^Department of Nutritional Sciences, University of Toronto, Toronto, Canada

^7^Department of Laboratory Medicine and Pathobiology, Faculty of Medicine, University of Toronto, Toronto, Canada

^8^Division of Endocrinology, The Hospital for Sick Children, Toronto, Canada

^9^Li Ka Shing Centre for Healthcare Analytics Research and Training, St. Michael’s Hospital, Toronto, Canada

^10^Preventative Cardiology, The Hospital for Sick Children, Toronto, Canada

^11^Children’s Hospital of Eastern Ontario Research Institute, Ottawa, Canada

# **Supplementary Methods**

## **Weight, Height/Length and zBMI**

To avoid overweighting visits that were not well-child physician visits, we randomly removed anthropometric observations (8.4% of routine physician visit observations) measured during routine physician visits that occurred close together in time,^1^ as follows: among children 0-6 months of age, we included routine physician visits that were at least one month apart; among children 6-18 months of age, we included routine physician visits that were at least 2 months apart; among children >18 months of age, we included routine physician visits that were at least 3 months apart.

We used the World Health Organization cutpoints of <-5.0 and >+5.0 SD-units to identify potentially implausible age- and sex-standardized z-scores of height, weight and BMI. ^2, 3^ ^4^Among potentially implausible anthropometric outliers, we compared these observations to values from a prior or subsequent visit within 2 years. If the comparison anthropometric value was within ± 2 SD-units of the outlying value we retained the extreme anthropometric observation. However, if the anthropometric value was >±2 SD-units and/or there were no prior or subsequent comparison visits available, we set the values to missing (149 observations excluded, which excluded 13 children).

## **Covariates**

Covariates were selected *a priori* based on review of the applicable or relevant literature, which has been shown to be superior to model selection procedures.^5^ Parents of participating children completed a Nutrition and Health Questionnaire adapted from the Canadian Community Health Survey.^6^ We categorized birthweight according to low (≥1kg <2.5kg), normal (≥2.5 and <4kg) and high (≥4kg) cut-offs.^7, 8^ Breastfeeding duration was determined from responses to the following questions as previously described^4^: (1) “Has your child ever been breastfed?”; (2) “Is your child currently breastfeeding?”; and (3) “At what age did you stop breastfeeding?”. Participants who had never breastfed were classified as having a duration of 0 months. Responses to the second and third question were used to determine duration of breastfeeding among children who were breastfed, with those who are currently breastfeeding classified as having duration equal to the child’s current age. A closed-ended question asked parents “what was your total family income before taxes last year?”, with response options given in $10,000 increments. Maternal education was categorized into 3 levels of completion: Public school; High school; College/University. Maternal ethnicity was self-reported in a closed-ended question and categorized into four groups: European; East Asian; Southeast/South Asian; Other. Parents reported history of hypertension as well as maternal hypertension during pregnancy. Maternal heights and weights were measured by research assistants at each clinic; maternal BMI was classified according to obesity status (≥30 vs <30 kg/m^2^).

# **Online Supplement References**

1. Bornhorst C, Tilling K, Russo P, Kourides Y, Michels N, Molnar D *et al.* Associations between early body mass index trajectories and later metabolic risk factors in European children: the IDEFICS study. *Eur J Epidemiol* 2016; **31**(5)**:** 513-25.

2. World Health Organization. *Physical status: the use and interpretation of anthropometry*: Geneva, 1995.

3. WHO. *WHO Child Growth Standards SAS igrowup package*: Geneva, 2011.

4. Eny KM, Chen S, Anderson LN, Chen Y, Lebovic G, Pullenayegum E *et al.* Breastfeeding duration, maternal BMI and birthweight are associated with differences in BMI growth trajectories in early childhood. *American Journal of Clinical Nutrition* 2018; **In Press**.

5. Harrell FE. Regression Modeling Strategies [electronic resource] : With Applications to Linear Models, Logistic and Ordinal Regression, and Survival Analysis In: *Springer Series in Statistics*, Second edn. Springer, 2015, pp 63-102.

6. Canada H. *Canadian Community Health Survey, Cycle 2.2, Nutrition (2004)*, 2006.

7. Kramer MS. Determinants of low birth weight: methodological assessment and meta-analysis. *Bull World Health Organ* 1987; **65**(5)**:** 663-737.

8. Jolly MC, Sebire NJ, Harris JP, Regan L, Robinson S. Risk factors for macrosomia and its clinical consequences: a study of 350,311 pregnancies. *Eur J Obstet Gynecol Reprod Biol* 2003; **111**(1)**:** 9-14.
