# Supplementary material for: Association of accelerated body mass index gain with repeated measures of blood pressure in early childhood
Source: Int J Obes (Lond). 2019 Apr 2;43(7):1354–62. doi: 10.1038/s41366-019-0345-9 (PMC6760600; doi:10.1038/s41366-019-0345-9)
Supplement: Supplementary file 3 — Supplementary Table 2 [file 41366_2019_345_MOESM3_ESM.docx]

# **Supplementary Table 1:** Estimated rates of zBMI gain during each period from birth to 36 months of age in Stage 1.

| Period (months) | Rate of zBMI  gain per month (SD) | 95% CI |
| --- | --- | --- |
| 0-3 mo | -0.12 (0.01) | -0.15, -0.09 |
| 3-18 mo | 0.06 (0.001) | 0.05, 0.06 |
| 18-36 mo | 0.01 (0.001) | 0.01, 0.02 |

Linear spline multilevel models were used to model rates of zBMI gain as previously described.^4^ Each child contributed a median of 7 repeated measures of zBMI (interquartile range = 3-9 measures) observations from birth to 36 months of age (n=4258, observations=26,843).
