# Supplementary material for: Association of accelerated body mass index gain with repeated measures of blood pressure in early childhood
Source: Int J Obes (Lond). 2019 Apr 2;43(7):1354–62. doi: 10.1038/s41366-019-0345-9 (PMC6760600; doi:10.1038/s41366-019-0345-9)
Supplement: Supplementary file 4 — Supplementary Table 3 [file 41366_2019_345_MOESM4_ESM.docx]

# **Supplementary Table 2: Proportion of children with repeated blood pressure measures, average age and levels of systolic and diastolic blood pressure on each occasion.**

| Visit | N | % With repeated measures | Average Age (months) | SBP | DBP |
| --- | --- | --- | --- | --- | --- |
| 1 | 2502 | -- | 44.0 | 87 | 56 |
| 2 | 1490 | 60% | 53.2 | 88 | 57 |
| 3 | 709 | 28% | 60.0 | 88 | 57 |
| 4 or more | 262 | 10% | 64.4 | 89 | 57 |
