# Supplementary material for: Association of accelerated body mass index gain with repeated measures of blood pressure in early childhood
Source: Int J Obes (Lond). 2019 Apr 2;43(7):1354–62. doi: 10.1038/s41366-019-0345-9 (PMC6760600; doi:10.1038/s41366-019-0345-9)
Supplement: Supplementary file 5 — Supplementary Table 4 [file 41366_2019_345_MOESM5_ESM.docx]

# **Supplementary Table 3**: Mean observed, mean predicted and mean difference in zBMI during each growth period.

| Growth  period  (months) | Mean Observed  zBMI  (95% CI) | Mean Predicted  zBMI  (95% CI) | Mean Difference  [observed – predicted]  (95% CI) |
| --- | --- | --- | --- |
| 0-3 | -0.52 (-2.82, 1.67) | -0.54 (-2.20, 1.10) | 0.018 (-1.05, 1.09) |
| 3-18 | -0.37 (-2.58, 1.92) | -0.36 (-2.10, 1.43) | -0.0070 (-1.12, 1.08) |
| 18-36 | 0.26 (-1.92, 2.33) | 0.26 (-1.40, 1.89) | 0.0055 (-0.98, 0.97) |

Mean predicted zBMIs from the unadjusted analyses for each growth period using piecewise linear mixed models to model mean zBMI growth rates (SD-units per month).
