# Supplementary material for: Association of accelerated body mass index gain with repeated measures of blood pressure in early childhood
Source: Int J Obes (Lond). 2019 Apr 2;43(7):1354–62. doi: 10.1038/s41366-019-0345-9 (PMC6760600; doi:10.1038/s41366-019-0345-9)
Supplement: Supplementary file 6 — Supplementary Figure 1 [file 41366_2019_345_MOESM6_ESM.docx]

# **Supplementary Table 4:** Association of zBMI at birth and rate of zBMI gain with repeated measures of BP (mm Hg) adjusted for concurrent zBMI.

|  | Systolic Blood Pressure | | Diastolic Blood Pressure | |
| --- | --- | --- | --- | --- |
| **Period (months)^a^** | **Β**^b,c^ | **95% CI** | **Β**^b,c^ | **95% CI** |
| **zBMI at birth**^d^ | 0.03 | (-0.21, 0.27) | 0.14 | (-0.09, 0.36) |
| **zBMI gain 0-3 m**^e^ | 0.55 | (0.28, 0.83) | 0.26 | (0.009, 0.50) |
| **zBMI gain 3-18 m** | 0.71 | (0.43, 1.0) | 0.42 | (0.17, 0.66) |
| **zBMI gain 18-36 m** | 0.41 | (-0.08, 0.90) | -0.04 | (-0.44, 0.36) |

^a^Each row shows results from four separate models.

^b^zBMI gain is sequentially conditioned on zBMI at birth and rate of zBMI gain in each period occurring prior to the current period.

^c^Adjusted for concurrent zBMI, age and height at time of BP measures, sex, family income, maternal (education, ethnicity, BMI, hypertension during pregnancy), parental history of hypertension, breastfeeding duration, visit type, and clinic.

^d^not adjusted for breastfeeding duration.

^e^Since we observed a deceleration of -0.12 zBMI-units per month in the first 3 months (Table S1), the interpretation of the positive effect estimate shows that a 1SD-unit slower rate of decrease in zBMI-units from 0-3 months was associated with higher BP (mmHg).
