# Supplementary material for: Association of accelerated body mass index gain with repeated measures of blood pressure in early childhood
Source: Int J Obes (Lond). 2019 Apr 2;43(7):1354–62. doi: 10.1038/s41366-019-0345-9 (PMC6760600; doi:10.1038/s41366-019-0345-9)
Supplement: Supplementary file 8 — Supplementary Figure 3 [file 41366_2019_345_MOESM8_ESM.docx]

# **Supplementary Figure 2: Stage 2 Flow chart of inclusion/exclusion criteria.**
