# Supplementary figures and images for: Association of accelerated body mass index gain with repeated measures of blood pressure in early childhood
Source: Int J Obes (Lond). 2019 Apr 2;43(7):1354–62. doi: 10.1038/s41366-019-0345-9 (PMC6760600; doi:10.1038/s41366-019-0345-9)

# **Supplementary Figure 3**

**
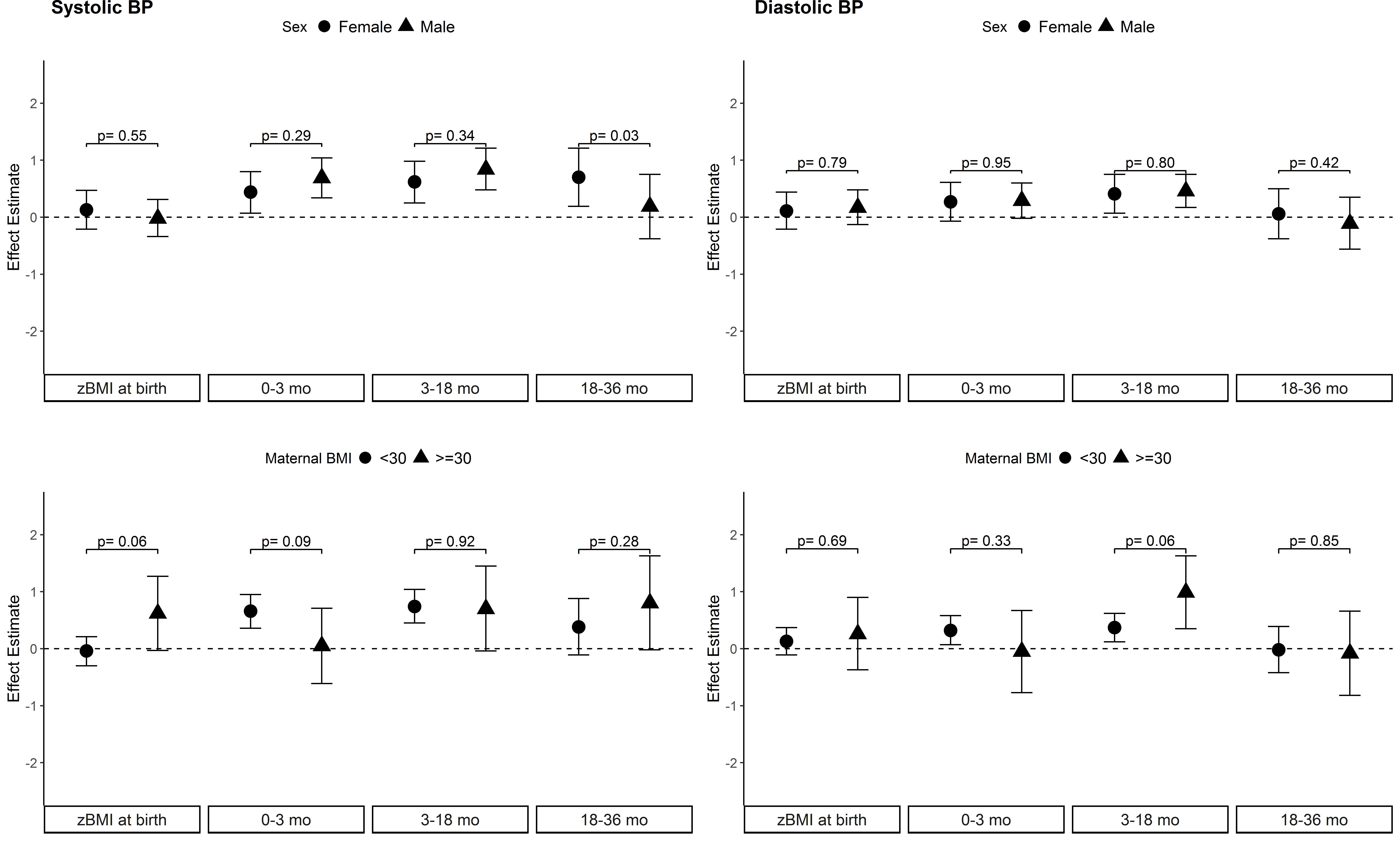
**

Supplement: Supplementary file 9 — Supplementary Figure 3 Legend [file 41366_2019_345_MOESM9_ESM.docx]
